# Growth Differentiation Factor 15 is a potential biomarker of therapeutic response for TK2 deficient myopathy

**DOI:** 10.1038/s41598-020-66940-8

**Published:** 2020-06-22

**Authors:** Cristina Dominguez-Gonzalez, Carmen Badosa, Marcos Madruga-Garrido, Itxaso Martí, Carmen Paradas, Carlos Ortez, Jordi Diaz-Manera, Andres Berardo, Jorge Alonso-Pérez, Selena Trifunov, Daniel Cuadras, Susana G. Kalko, Cora Blázquez-Bermejo, Yolanda Cámara, Ramon Martí, Fabiola Mavillard, Miguel A. Martin, Julio Montoya, Eduardo Ruiz-Pesini, Joan Villarroya, Raquel Montero, Francesc Villarroya, Rafael Artuch, Michio Hirano, Andrés Nascimento, Cecilia Jimenez-Mallebrera

**Affiliations:** 1 Neurology Department, Neuromuscular Disorders Unit, 12 de Octubre Hospital, Madrid, Spain; 2 Research Institute i+12, 12 de Octubre Hospital, Madrid, Spain; 3 Biomedical Network Research Centre on Rare Diseases (CIBERER), Instituto de Salud Carlos III, Madrid, Spain; 4 Neuromuscular Unit, Neuropediatrics Department, Institut de Recerca Sant Joan de Déu, Hospital Sant Joan de Déu, Barcelona, Spain; 5 Neuromuscular Disorders Unit, Neuropediatrics Department, Instituto de Biomedicina de Sevilla, Hospital Universitario Virgen del Rocío, Consejo Superior de Investigaciones Científicas, University of Seville, Seville, Spain; 6 Neuropediatrics Department, Hospital Universitario Donostia, San Sebastian, Spain; 7 Neuromuscular Disorders Unit, Neurology Department, Instituto de Biomedicina de Sevilla, Hospital Universitario Virgen del Rocío, Consejo Superior de Investigaciones Científicas, University of Seville, Seville, Spain; 8 Biomedical Network Research Centre on Neurodegenerative Diseases (CIBERNED), Instituto de Salud Carlos III, Madrid, Spain; 9 Neuromuscular Diseases Unit, Hospital de la Santa Creu i Sant Pau, Universitat Autònoma de Barcelona, Barcelona, Spain; 10 Department of Neurology, Columbia University Medical Center, New York, USA; 11 Statistics Unit, Fundación Sant Joan de Déu, Barcelona, Spain; 12 Moebius Research Ltd, Systems Biomedicine, London, UK; 13 Research group on Neuromuscular and Mitochondrial Diseases, Vall d’Hebron Institut de Recerca, Universitat Autònoma de Barcelona, Barcelona, Spain; 14 Departmento de Bioquímica y Biología Molecular, Universidad de Zaragoza, Instituto de Investigación Sanitaria de Aragón (IIS-Aragón), Zaragoza, Spain; 15 Biochemistry and Molecular Biology Department, Institute of Biomedicine (IBUB), Institut de Recerca Sant Joan de Déu, University of Barcelona, Barcelona, Spain; 16 Biomedical Network Research Centre on Obesity and Nutrition (CIBEROBN), Instituto de Salud Carlos III, Madrid, Spain; 17 Clinical Biochemistry Department, Institut de Recerca Sant Joan de Déu, Hospital Sant Joan de Déu, Barcelona, Spain; 18 Genetics Department, Faculty of Biology, University of Barcelona, Barcelona, Spain

**Keywords:** Genetics, Neuroscience, Biomarkers, Diseases, Neurology

## Abstract

GDF-15 is a biomarker for mitochondrial diseases. We investigated the application of GDF-15 as biomarker of disease severity and response to deoxynucleoside treatment in patients with thymidine kinase 2 (TK2) deficiency and compared it to FGF-21. GDF-15 and FGF-21 were measured in serum from 24 patients with TK2 deficiency treated 1–49 months with oral deoxynucleosides. Patients were grouped according to age at treatment and biomarkers were analyzed at baseline and various time points after treatment initiation. GDF-15 was elevated on average 30-fold in children and 6-fold in adults before the start of treatment. There was a significant correlation between basal GDF-15 and severity based on pretreatment distance walked (6MWT) and weight (BMI). During treatment, GDF-15 significantly declined, and the decrease was accompanied by relevant clinical improvements. The decline was greater in the paediatric group, which included the most severe patients and showed the greatest clinical benefit, than in the adult patients. The decline of FGF-21 was less prominent and consistent. GDF-15 is a potential biomarker of severity and of therapeutic response for patients with TK2 deficiency. In addition, we show evidence of clinical benefit of deoxynucleoside treatment, especially when treatment is initiated at an early age.

## Introduction

Mitochondrial DNA depletion and deletion syndrome 2 (MTDPS2) is caused by mutations in the nuclear gene *TK2* that encodes thymidine kinase 2 (*TK2*) which is necessary for mitochondrial DNA replication and maintenance^1^. TK2 deficiency manifests predominantly as a myopathy and includes extremely severe and rapidly progressive early-onset forms with survival of less than two years, to milder forms with late or very late-onset, and a slower rate of progression, but with almost invariable respiratory involvement than shortens life expectancy^2–4^.

A new therapy for TK2 deficiency is under investigation, based on administration of oral deoxynucleosides. The rationale is to induce the synthesis of pyrimidine deoxynucleotides (dTTP and dCTP) via alternative enzyme pathways by means of supplementation with their precursors. Although some patients started with taking oral deoxynucleotides (dTMP and dCMP), after the demonstration *in vitro* and *in vivo*^5,6^ that deoxynucleosides are the active agents, the therapy was changed to deoxynucleosides (dThd and dCtd). The efficacy of oral deoxynucleoside treatment was demonstrated in pre-clinical studies^7,8^ and in a group of 16 patients treated under a compassionate use program^9^. Without any major side effect, the therapy had striking effects on early-onset severe myopathy patients, such as improvement in muscle strength, reduction or discontinuation of mechanical ventilation and gastrostomy feeding, and regaining the ability to walk. The patients also showed considerable functional improvements according to motor outcome measures in childhood-onset cases and at least stabilization in late-onset ones^9^.

Biomarkers are playing an increasingly important role in drug development and treatment implementation^10^. In addition to contributing to a better understanding of diseases, biomarkers provide more sensitive and specific means of diagnosing and ways to determine responses to new treatments. In this way, they help to streamline clinical trial efficacy and reduce uncertainty in regulatory decision-making, thus accelerating drug approval and drug access for patients.

According to BEST (The Biomarkers, EndpointS and other Tools, FDA-NIH Biomarker Working Group, https://www.ncbi.nlm.nih.gov/books/NBK326791/) a monitoring biomarker is a biomarker which is measured serially for assessing the status of a disease or for evidence of an effect of a medical product. A pharmacodynamics/response biomarker is used to show that a biological response has occurred in an individual who has been exposed to a medical product.

Growth Differentiation Factor 15 (GDF-15) is a cytokine that is induced in response to various stimuli and in different pathological situations where it is associated with disease progression and negative prognosis^11–13^. We previously demonstrated that GDF-15 is a valuable circulating diagnostic biomarker for mitochondrial diseases including mitochondrial DNA depletion and deletion syndromes, MELAS and KSS^14–16^. Fibroblast Growth Factor 21 (FGF-21) has also been reported to be elevated in a range of mitochondrial diseases, particularly in those with muscle involvement^17^. Determination of circulating GDF-15 and FGF-21 has greatly aided the diagnosis of mitochondrial pathologies since their sensitivity is significantly higher than other conventional biomarkers such as venous lactate level^16,18^.

The objective of this study was to explore the application of circulating GDF-15 as a biomarker of disease severity and prognosis as well as to monitor response to deoxynucleosides treatment in patients with TK2 deficiency, and compare it with FGF-21. Furthermore, since GDF-15 has been linked to inflammation in some diseases^13,19^ we also investigated circulating levels of inflammatory cytokines.

## Results

### Patients

The 26 patients included span the spectrum of phenotypes associated with TK2 deficiency^4^. Pre-treatment data are summarized in Table 1. Patients P25 and P26 had to stop treatment soon after initiation because of an increase in liver enzymes. They were not further analyzed. Follow-up data of P1 to P24 are available in Tables 2 and 3.

**Table 1:** Clinical, biochemical and molecular characteristics of patients, before treatment.

CLINICAL FORM	ID	SEX	AGE AT ONSET	BMI	WALK INDEPENDENTLY (Y/N)	MECHANICAL VENTILATION (Y/N)	PEG (Y/N)	GENOTYPE (Allele 1/Allele 2)	DEPLETION (relative to normal)	MULTIPLE DELETIONS (Y/N)	CK (UI/l)	BASAL GDF-15 (pg/mL)	BASAL FGF-21 (pg/mL)
GROUP 1 (started treatment <16)	1	F	23 m	<P3	N	N	N	p.Lys202del/p.Asp177Tyr	25%	N	1183	15.000	1373
	2	M	30 m	<P3	N	Y	NGT	p.His121Asn/p.His12Asn	50%	Y	538	4608	402
	3	M	17 m	P3	N	Y	NGT	p.Tyr208Cys/p.Arg130Trp	15%	N	148	14756	3013
	4	M	15 m	<P3	N	Y	N	p.His121Asn/p.Arg192Lys	13%	N	284	ND	ND
	5	M	13 m	<P3	N	Y	NGT	p.Thr108Met/Leu215Pro	ND	ND	872	21641	4088
	6	M	13 m	<P3	N	Y	NGT	p.Thr108Met/Leu215Pro	ND	ND	949	22767	3244
	7	M	13 m	<P3	N	N	N	p.Lys85*/p.Pro154Leu	ND	ND	921	27066	7531
	8	M	24 m	ND	ND	ND	ND	p.Thr108Met/p.Thr108Met	ND	ND	285	4597	ND
	9	M	31 m	ND	ND	ND	ND	p.Thr108Met/p.Thr108Met	8%	ND	626	6825	ND
	10	F	3 y	P3	Y	Y	NGT	p.Asn58Ser/p.Asn58Ser	ND	ND	1789	4797	809
	11	M	3 y	P50	Y	N	N	p.Asn58Ser/p.Asn58Ser	ND	ND	1036	2641	398
	12	F	2 y	P50	Y	N	N	p.His121Asn/p.Arg183Trp	ND	ND	460	3686	786
	13	F	2 y	P50	Y	N	N	p.His121Asn/p.Arg183Trp	ND	ND	945	3841	471
	14	M	3 y	P3	N	Y	N	p.His121Asn/p.Arg183Trp	ND	ND	184	5006	1057
	15	F	6 y	<P3	Y	N	N	p.Asn58Ser/p.Asn58Ser	ND	ND	742	2781	595
GROUP 2 (started treatment >16)	16	F	> 12 y	13.86	Y	Y	N	p.Thr108Met/p.Thr108Met	17%	Y	2435	2423	666
	17	M	50 y	27.7	Y	Y	N	p.Lys202del/p.Lys202del	66%	Y	357	1529	252
	18	F	30 y	27.12	Y	Y	N	p.Lys202del/p.Lys202del	60%	Y	294	1695	353
	19	F	20 y	17.79	Y	Y	Y	p.Thr108Met/p.Thr108Met	39%	Y	303	2439	197
	20	F	60 y	26.01	Y	Y	N	p.Lys202del/p.Lys202del	ND	ND	647	2483	897
	21	M	30 y	23.7	Y	N	N	p.Lys202del/p.Lys202del	ND	Y	350	1640	185
	22	F	5 y	26.17	Y	Y	N	p.Thr108Met/p.Thr108Met	ND	Y	548	2149	943
	23	F	30 y	ND	Y	Y	N	p.Thr108Met/p.Thr108Met	53%	Y	233	1261	190
	24	F	20 y	20,5	Y	Y	GT	p.Lys202del/p.Ala420Val	ND	ND	1142	1979	179
	25	F	50 Y	28,7	Y	Y	N	p.Lys202del/p.Lys202del	ND	Y	405	ND	ND
	26	F	14 Y	24,5	Y	Y	N	p.Thr108Met/p.Thr108Met	19%	Y	425	ND	ND

BMI, body-mass index; CK, creatine kinase; F, female; GDF-15, differentiation growth factor 15; M, male; m, months; ND, not determined; NGT, nasogastric tube; N, No; P, percentile; PEG, percutaneous endoscopic gastrostomy; y, years; Y, yes.

**Table 2:** Outcome measures, Group 1 (age at treatment initiation <16 years).

ID		1	2	3	4	5	6	7	8	9	10	11	12	13	14	15
AGE OF ONSET AGE AT INITIATION OF TREATMENT CURRENT AGE TREATMENT DOSE (mg/kg/d)		23 m	30 m	17 m	15 m	13 m	13 m	13 m	24 m	31 m	36 m	36 m	24 m	24 m	36 m	6 y
		27 m	10 y	30 m	2.5 y	15 m	15 m	14 m	33 m	6 y	14 y	12 y	3 y	3 y	7 y	9 y
		5 y	13 y	7 y	7 y	21 m	21 m	24 m	3 y	7 y	16 y	13 y	5 y	5 y	9 y	10 y
		400	300	400	400	400	400	400	400	400	400	400	400	400	400	400
CK	BASAL	1183	538	148	284	872	940	921	285	626	1789	759	460	945	184	742
	0–6 Months	ND	316	ND	ND	649	561	275	133	175	235	243	606	857	164	269
	6–12 Months	114	ND	68	132	ND	ND	164	130	165	273	290	173	87	111	195
	12–24 Months	55	712	ND	116	ND	ND	ND	ND	ND	ND	ND	ND	ND	ND	ND
	>24 Months	87	285	ND	61	ND	ND	ND	ND	ND	ND	ND	ND	ND	ND	ND
GDF-15 (pg/mL)	BASAL	15000	4608	14756	ND	21641	22767	27066	ND	ND	4797	2641	3686	3841	5006	2781
	0–6 Months	1200	762	ND	ND	6628	2174	588	4597	6825	4753	3105	ND	2635	5180	2249
	6–12 Months	497	ND	5093	2021	ND	ND	ND	396	369	ND	ND	341	330	440	345
	12–24 Months	392	2084	594	255	ND	ND	ND	ND	ND	ND	ND	ND	ND	ND	ND
	>24 Months	288	631	ND	284	ND	ND	ND	ND	ND	ND	ND	ND	ND	ND	ND
FGF-21 (pg/mL)	BASAL	1373	402	3013	ND	4088	3245	7531	ND	ND	809	398	786	471	1057	595
	0–6 Months	72	228	ND	ND	1445	776	208	583	743	769	1090	ND	351	1077	417
	6–12 Months	31	ND	2170	415	ND	ND	ND	ND	ND	ND	ND	42	<LOQ	<LOQ	34
	12–24 Months	30	689	603	51	ND	ND	ND	ND	ND	ND	ND	ND	ND	ND	ND
	>24 Months	37	134	ND	100	ND	ND	ND	ND	ND	ND	ND	ND	ND	ND	ND
BMI	BASAL	<P3	<P3	P3	P1	13,2	13,5	12,7	ND	ND	15,3	20,2	13,5	16	11,5	10,5
	0–6 Months	ND	9.6	ND	ND	12,8	13,2	12,9	ND	ND	16,1	19,05	15,02	15,08	11	10,7
	6–12 Months	17.2	ND	16.02	12.75	ND	ND	ND	ND	ND	ND	ND	ND	ND	ND	ND
	12–24 Months	16.3	20	ND	ND	ND	ND	ND	ND	ND	ND	ND	ND	ND	ND	ND
	>24 Months	14.9	25.3	ND	ND	ND	ND	ND	ND	ND	ND	ND	ND	ND	ND	ND
6MWT	BASAL	0	ND	ND	ND	ND	ND	ND	ND	420	75	342	250	272	0	307
	0–6 Months	150	ND	ND	ND	ND	ND	ND	ND	531	350	354	ND	ND	ND	364
	6–12 Months	250	ND	ND	ND	ND	ND	ND	ND	512	379	375	394	368	15	ND
	12–24 Months	391	ND	ND	ND	ND	ND	ND	ND	ND	ND	ND	ND	ND	ND	ND
	>24 Months	477	ND	ND	ND	ND	ND	ND	ND	ND	ND	ND	ND	ND	ND	ND
EK2	BASAL	ND	26	30	28	ND	ND	ND	ND	ND	ND	ND	ND	ND	ND	ND
	0–6 Months	ND	ND	ND	ND	ND	ND	ND	ND	ND	ND	ND	ND	ND	ND	ND
	6–12 Months	ND	9	21	14	ND	ND	ND	ND	ND	ND	ND	ND	ND	ND	ND
	12–24 Months	ND	ND	ND	12	ND	ND	ND	ND	ND	ND	ND	ND	ND	ND	ND
	>24 Months	ND	13	ND	11	ND	ND	ND	ND	ND	ND	ND	ND	ND	ND	ND
NSAA	BASAL	4	ND	ND	ND	ND	ND	ND	20	27	ND	ND	ND	ND	ND	ND
	0–6 Months	6	ND	ND	ND	ND	ND	ND	ND	33	ND	ND	ND	ND	ND	ND
	6–12 Months	20	ND	ND	ND	ND	ND	ND	31	33	ND	ND	ND	ND	ND	ND
	12–24 Months	26	ND	ND	ND	ND	ND	ND	ND	ND	ND	ND	ND	ND	ND	ND
	>24 Months	ND	ND	ND	ND	ND	ND	ND	ND	ND	ND	ND	ND	ND	ND	ND
HMFS	BASAL	ND	ND	ND	ND	ND	ND	ND	ND	ND	21	35	38	38	17	38
	0–6 Months	ND	ND	ND	ND	ND	ND	ND	ND	ND	30	33	ND	ND	ND	39
	6–12 Months	ND	ND	ND	ND	ND	ND	ND	ND	ND	36	37	39	39	23	ND
	12–24 Months	ND	ND	ND	ND	ND	ND	ND	ND	ND	ND	ND	ND	ND	ND	ND
	>24 Months	ND	ND	ND	ND	ND	ND	ND	ND	ND	ND	ND	ND	ND	ND	ND
HMFSE	BASAL	ND	ND	ND	ND	ND	ND	ND	ND	ND	26	51	51	53	19	62
	0–6 Months	ND	ND	ND	ND	ND	ND	ND	ND	ND	48	49	ND	ND	ND	65
	6–12 Months	ND	ND	ND	ND	ND	ND	ND	ND	ND	57	55	65	65	27	ND
	12–24 Months	ND	ND	ND	ND	ND	ND	ND	ND	ND	ND	ND	ND	ND	ND	ND
	>24 Months	ND	ND	ND	ND	ND	ND	ND	ND	ND	ND	ND	ND	ND	ND	ND
CHOP INTEND	BASAL	ND	ND	ND	ND	42	45	17	ND	ND	ND	ND	ND	ND	ND	ND
	0–6 Months	ND	ND	ND	ND	45	54	64	ND	ND	ND	ND	ND	ND	ND	ND
	6–12 Months	ND	ND	ND	ND	ND	ND	ND	ND	ND	ND	ND	ND	ND	ND	ND
	12–24 Months	ND	ND	ND	ND	ND	ND	ND	ND	ND	ND	ND	ND	ND	ND	ND
	>24 Months	ND	ND	ND	ND	ND	ND	ND	ND	ND	ND	ND	ND	ND	ND	ND
FVC (%)	BASAL	ND	13	ND	ND	ND	ND	ND	ND	ND	ND	ND	ND	ND	ND	38
	0–6 Months	ND	ND	ND	ND	ND	ND	ND	ND	ND	19	50	ND	ND	ND	66
	6–12 Months	ND	22	ND	ND	ND	ND	ND	ND	ND	35	70	ND	ND	71	ND
	12–24 Months	ND	ND	ND	ND	ND	ND	ND	ND	ND	ND	ND	ND	ND	ND	ND
	>24 Months	ND	26	ND	ND	ND	ND	ND	ND	ND	ND	ND	ND	ND	ND	ND

CK, creatine kinase; EK, Egen Klassifikation; HMFS, Hammersmith Motor Functional Scale; HMFSE, Hammersmith Motor Functional Scale Expanded; 6MWT, 6-minute walk test; m, months; ND, not determined; NSAA, North Star Ambulatory Assessment; y, years.

**Table 3:** Outcome measures, Group 2 (age at treatment initiation >16 years).

ID		16	17	18	19	20	21	22	23	24
AGE OF ONSET AGE AT INITIATION OF TREATMENT CURRENT AGE TREATMENT DOSE (mg/kg/d)		>12 y	50 y	30 y	20 y	60 y	36 y	5 y	30 y	20 y
		30 y	58 y	59 y	31 y	74 y	60 y	35 y	46 y	57 y
		34 y	62 y	62 y	34 y	75 y	60 y	36 y	46 y	60 y
		320	300	400	400	400	400	350	300	400
CK (UI/l)	BASAL	2435	525	294	303	647	402	548	188	1142
	0–6 Months	ND	ND	123	322	ND	264	107	ND	1195
	6–12 Months	393	ND	258	292	105	ND	142	ND	1348
	12–36 Months	330	351	108	110	ND	ND	ND	ND	ND
	>36 Months	561	459	185	467	89	ND	ND	ND	ND
GDF-15 (pg/mL)	BASAL	2423	1529	1695	2439	2483	1640	2149	1261	1980
	0–6 Months	ND	ND	ND	ND	1364	1357	473	486	1052
	6–12 Months	ND	ND	ND	ND	1320	ND	413	ND	1011
	12–36 Months	464	782	704	1110	1099	ND	ND	ND	ND
	>36 Months	480	1137	753	783	ND	ND	ND	ND	ND
FGF-21 (pg/mL)	BASAL	666	252	353	197	897	185	943	190	179
	0–6 Months	ND	ND	ND	ND	732	135	47	ND	113
	6–12 Months	ND	ND	ND	ND	344	ND	ND	ND	155
	12–36 Months	1222	944	245	915	ND	ND	ND	ND	ND
	>36 Months	632	1025	421	ND	ND	ND	ND	ND	ND
BMI	BASAL	13.86	27.7	27.12	17.79	26.01	23.7	26.17	ND	20,8
	0–6 Months	ND	ND	ND	19,06	25	23.6	25.4	ND	21
	6–12 Months	ND	ND	ND	21,09	27,6	ND	ND	ND	20,4
	12–36 Months	16.06	ND	26.3	21.23	ND	ND	ND	ND	ND
	>36 Months	ND	ND	26.9	18,63	ND	ND	ND	ND	ND
6MWT	BASAL	532	475	386	390	345	ND	368	413	225
	0–6 Months	ND	500	355	437	ND	ND	435	515	251
	6–12 Months	ND	ND	400	474	ND	ND	450	ND	228
	12–36 Months	550	435	354	369	ND	ND	ND	ND	ND
	>36 Months	600	442	450	476	ND	ND	ND	ND	ND
NSAA	BASAL	ND	ND	26	21	29	ND	16	30	ND
	0–6 Months	30	24	26	21	ND	ND	25	31	ND
	6–12 Months	ND	ND	29	23	ND	ND	26	ND	ND
	12–36 Months	29	27	32	28	ND	ND	ND	ND	ND
	>36 Months	30	ND	31	29	ND	ND	ND	ND	ND
PIM (%)	BASAL	25	40	49.9	5	59	ND	37	31	ND
	0–6 Months	ND	ND	40.2	ND	33,9	ND	37,8	ND	ND
	6–12 Months	29.5	ND	53	24	47.3	ND	ND	ND	ND
	12–36 Months	21.4	38.6	49.9	ND	39,8	ND	ND	ND	ND
	>36 Months	ND	28	53	27	ND	ND	ND	ND	ND
FVC (%)	BASAL	46.5	45	71.8	26	69	78	53.2	63.7	ND
	0–6 Months	ND	51.4	79.5	33	66,5	82.6	62	64	16
	6–12 Months	50.3	ND	75	23,7	78,9	ND	ND	ND	17
	12–36 Months	46.7	46.7	77.7	32,2	83,4	ND	ND	ND	ND
	>36 Months	50.5	43.6	72	34,8	ND	ND	ND	ND	ND

ND, not determined; CK, creatine kinase; y, years; m, months; NA, not available, GDF-15, growth differentiation factor 15; FGF-21, fibroblast growth factor 21; BMI, body mass index; 6MWT, six-minute walk test; NSAA, north star ambulatory assessment; PIM, maximal inspiratory pressure; FVC, forced vital capacity.

The 24 treated patients were grouped according to age at treatment initiation: group 1 (the paediatric group) started treatment before 16 years of age (P1 to P15), and group 2 (the adult group) started treatment after this age (P16 to P24). This grouping allowed us to compare GDF-15 and FGF-21 serum levels with aged-matched controls and takes into account the observation that age-related metabolic changes contribute to the efficacy of deoxynucleoside therapy^8^.

Group 1 consisted of 15 children (five female and ten male), six of them with early-onset and very rapid progression (P1, P3, P4, P5, P6, and P7) and nine with childhood-onset and an intermediate disease course (P2 and P8-P15). Group 2 consisted of nine patients (seven female and two male) and included one patient with childhood-onset (P22) and eight patients with late-onset forms (P16 to P21 and P23 to P24). To assess disease progression, patients were compared with other patients with similar clinical phenotypes based on previous publications^2,4,9^. The follow-up time ranged from 1 to 49 months for patients in group 1 and from 1 to 46 months for patients in group 2. A summary of the age of onset and treatment initiation for each group is provided in Table 4.

**Table 4:** Summary of descriptive statistics.

Group 1	Mean	SE	Range
Age onset	27.1 m	3.8	13–72 m
Age treatment start	62.1 m	12.6	14–168 m
Basal GDF-15 (pg/mL)	10716	2579	2641–27066
Basal FGF-21 (pg/mL)	1981	702	402–7531
**Group 2**			
Age onset	29.2 y	5.3	5–60 y
Age treatment start	50 y	4.3	30–74 y
Basal GDF-15 (pg/mL)	1955	149	1261–2483
Basal FGF-21 (pg/mL)	429	106	179–943

m, months; y, years; SE, standard error of the mean.

### Change of serum GDF-15 and FGF-21 over time in treated patients

We previously described the baseline levels of GDF-15 in a healthy pediatric population^15^. For the purposes of this study, we analyzed GDF-15 serum levels in 13 adult healthy controls (age range 23–56 years) and found that the mean GDF-15 concentration was 323 pg/mL (SE 19.7; range 232–460 pg/mL) which is comparable to the mean values in the pediatric (age range 1 month to 18 years) control population (350 pg/mL; SE 20.7, range 155–584) and overlaps with values reported elsewhere^20^. Baseline levels were available from 12 participants in group 1 and nine in group 2. The mean values and ranges for GDF-15 and FGF-21 for both groups of patients are summarised in Table 4. GDF-15 levels were increased on average by 30-fold in group 1 (mean 10716 pg/mL; SE 2579; range 2641–27066), and by six-fold in group 2 (mean 1955 pg/mL; SE 149; range 1261–2483), relative to the mean values in the control groups. If we only take into account the five patients from group 1 with the most severe disease form for whom we have basal measurements, the average GDF-15 levels were 60-fold increased over the value in the paediatric control group (mean 20246 pg/mL; SE 2371; range 14756–27066).

For FGF-21, average baseline levels were elevated by 25-fold in group 1 (mean 1981 pg/mL; SE 702; range 402–731) and by six-fold in group 2 (mean 429 pg/mL; SE 106; range 179–943), relative to control values^16^. Similarly to GDF-15, the average basal FGF-21 value almost doubled if we only took into account the most severe patients from group 1 (Table 4).

**Figure 1:**
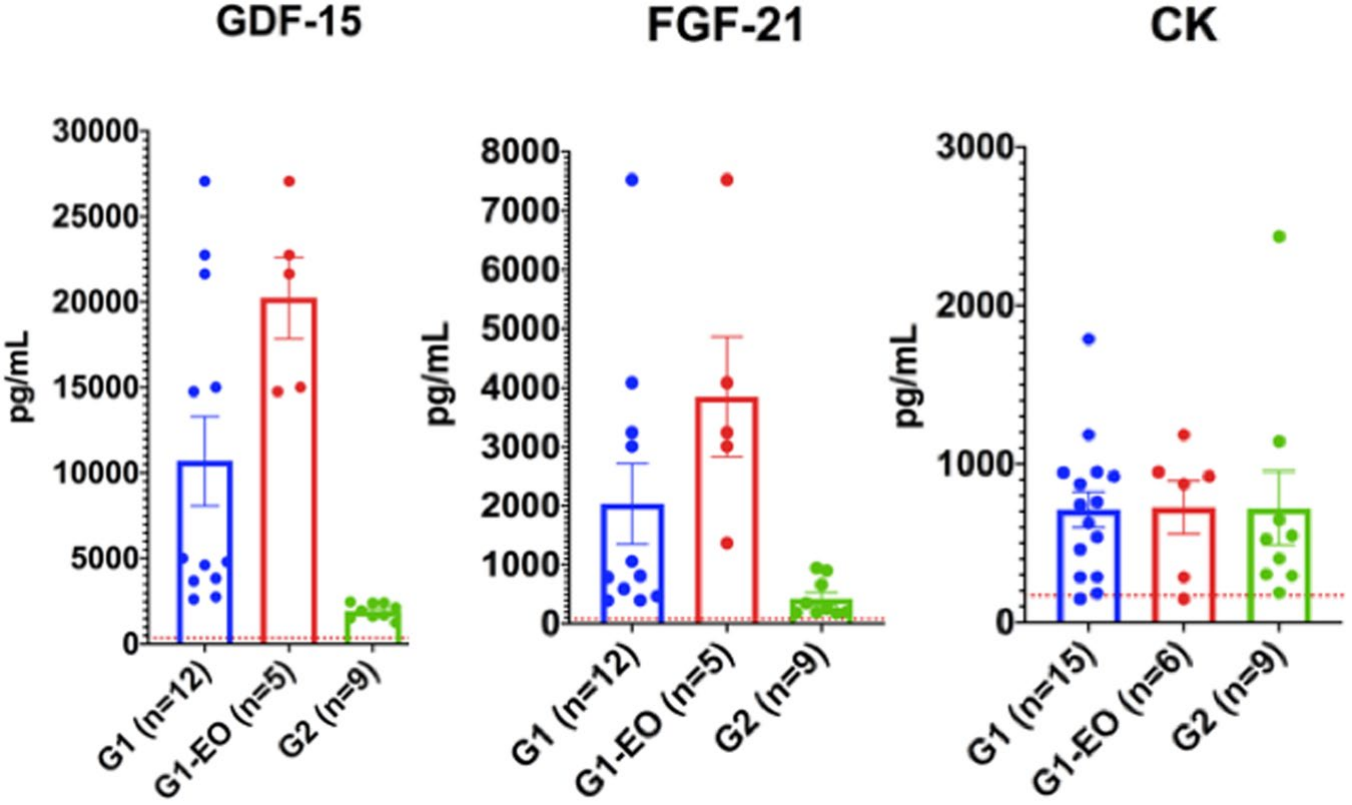
Pre-treatment GDF-15 and FGF-21 serum levels are associated with disease severity. Histograms representing the basal (before treatment) serum levels (mean and SEM) of GDF-15, FGF-21 and creatine kinase (CK) in patients from Group 1 (G1), those patients from Group 1 with the early onset and the most severe phenotype (G1-EO) and patients in Group 2 (G2). Dotted lines represent control values.

Thus, both GDF-15 and FGF-21 basal serum levels are associated with the severity of the phenotype. This was, however, not the case for CK since its basal levels were comparable regardless of the severity of the phenotype (Fig. 1).

**Figure 2:**
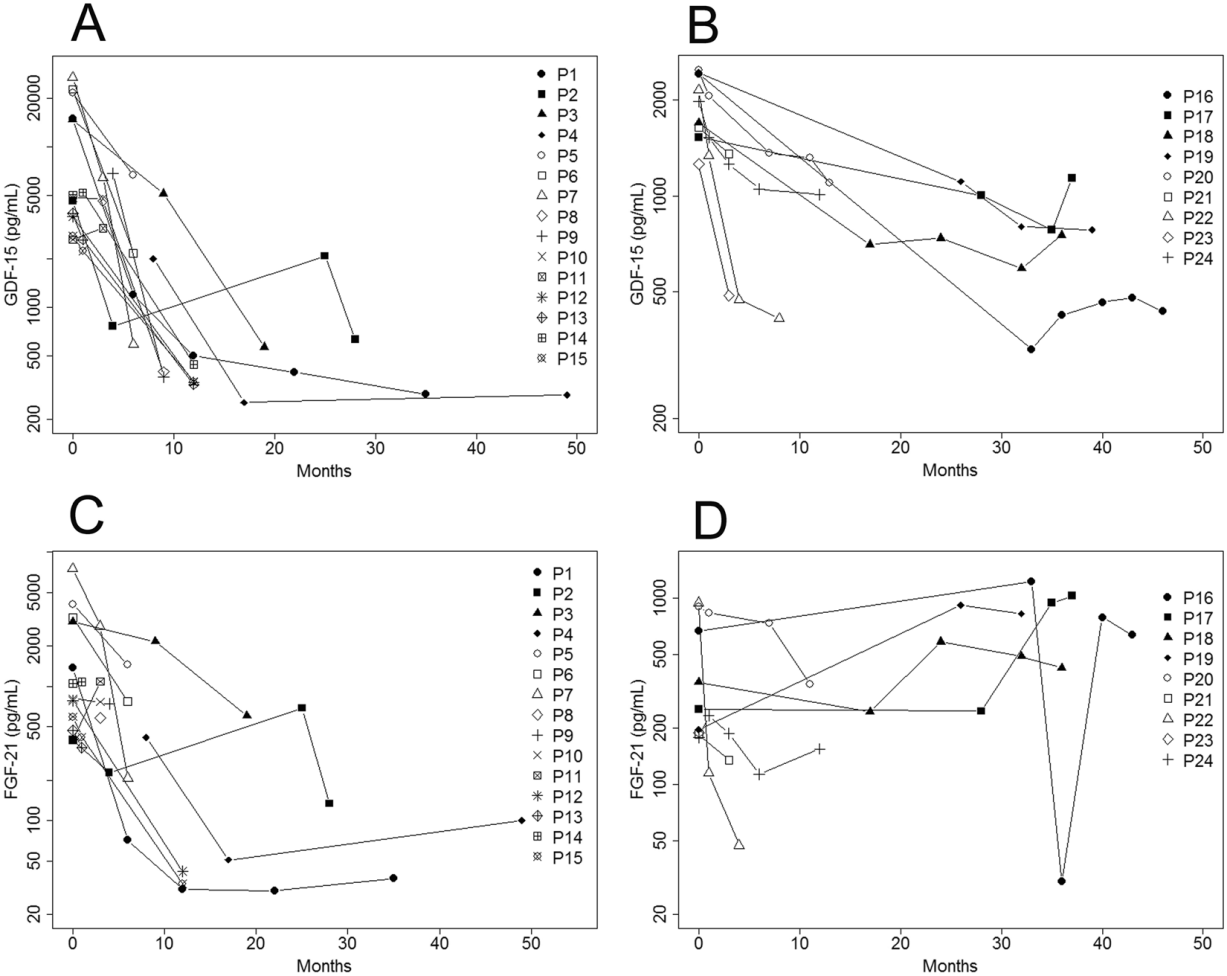
GDF-15 levels decreased during treatment with deoxynucleosides. Development of GDF-15 (**A**,**B**) and FGF-21 (**C**,**D**) circulating concentrations (pg/mL) over time in group 1 (left) and group 2 (right) patients.

The development of serum GDF-15 values at different times after the initiation of treatment is represented in Fig. 1. We applied a linear mixed model using the log-transformed GDF-15 concentration levels and found a significant decrease over time in patients in both group 1 (p < 0.0001) and group 2 (p = 0.0082) (Fig. 2A,B). FGF-21 followed a similar trend, although not significant, for group 1 (p = 0.062) but this trend was not present in group 2 (Fig. 2C,D).

Because the time points at which GDF-15 levels were measured are not uniform across patients and to homogenize the data we grouped the measurements at regular intervals corresponding to the follow-up intervals for each group (Supp. Fig. 1). We observed that in group 1 GDF-15 values decreased steadily over time, particularly between 0 and 24 months and that the difference between intervals was statistically significant (Kruskal-Wallis chi-squared p < 0.001). After 12 months of treatment, most values were very close or below the normal threshold (550 pg/mL) with the exception of P2.

In patient P2 the GDF-15 concentration already decreased to almost normal levels after only four months of treatment. For this patient the next measurement was performed after two years of treatment and a few days after the nucleoside treatment dose was reduced to 300 mg/Kg (and remained at this dose) because of diarrhea, and at this point GDF-15 was increased (to half the baseline level). Three months later, GDF-15 had almost returned to normal levels in P2. We do not know when exactly the increase in GDF-15 occurred and whether it happened before or during the few days interval between the change of dose and the measurement.

When we analysed FGF-21 levels, we found significant differences between the intervals (p = 0.01) although after 12 months of treatment half of the patients still had levels above the normal threshold (Supp. Fig. 1).

In the group that started treatment at an older age (>16 years-old), consisting mainly of patients with a late-onset phenotype, biomarker determinations spanned a longer period of time (0 to >36 months). Although the differences in GDF-15 levels between the different intervals were significant (Kruskal-Wallis chi-squared p = 0.003), levels appeared to decrease mainly during the first 12 months of treatment and then remained stable (Supp. Fig. 1). In contrast, there was no clear trend for FGF-21 and in some patients levels even increased with treatment. In this case, the difference between intervals was not significant (Supp. Fig. 1).

We calculated the rate of decline of GDF-15 and FGF-21 levels relative to the basal levels in the same time intervals in the paediatric and adult patients for whom we had measurements before treatment (% change at time T = log GDF-15 at T-log GDF-15 at T0/log GDF-15 T0 x 100). The rate of decline increased with time in group 1 for GDF-15 (−12.5% between 0 and 6 months, −26.7% between 6 and 12 months, −26.9% between 12 and 24 months and 32.3% after 24 months) and FGF-21 (−11.5%, −36.3%, −21.3% and −34.2% in the same intervals) whereas it was rather uniform in group 2 for GDF-15 (−10.3% between 0 and 6 months, −12.8% between 6 and 12 months, −12.5% between 12 and 36 months, and −12.6% after 36 months) and for FGF-21 (−15.4%, −8.4%, −14% and −9.1% for the same time intervals).

### Change of serum GDF-15 over time in patients that stopped treatment

Patients P25 and P26 stopped treatment because their liver enzymes had increased. P25 received treatment for three months, then stopped for three months, and then took nucleosides for one more month before stopping treatment completely. We do not have baseline levels for P25. The first GDF-15 measurement, performed after 6 months without treatment, was elevated (1833 pg/mL). One month later, GDF-15 levels had decreased to 1489 pg/mL, two months later (9 months after stopping the treatment completely) GDF-15 went up to 1675 pg/mL. Then they remained elevated (1657 pg/mL) even after 18 months of stopping the treatment when the last measurement was taken.

P26 received treatment for three months, stopped for four months, and then restarted treatment for one more month before stopping completely. GDF-15 levels were measured nearly three months after treatment interruption and were then mildly elevated (688 pg/ml). Then, in this patient, they remained stable over the next four months (including one month with treatment) to 639 pg/mL. Three months later (10 months after the first treatment interruption) it had risen to 818 pg/ml. From then onwards, levels continued to rise (1301 pg/mL after 13 months of treatment interruption) to reach 1656 pg/mL at the last measurement, 16 months after treatment interruption.

Thus, the trend of GDF-15 in the absence of treatment appears to reflect the disease course in these two patients, P25 has remained clinically stable whilst P26 has a more progressive form of the disease.

**Figure 3:**
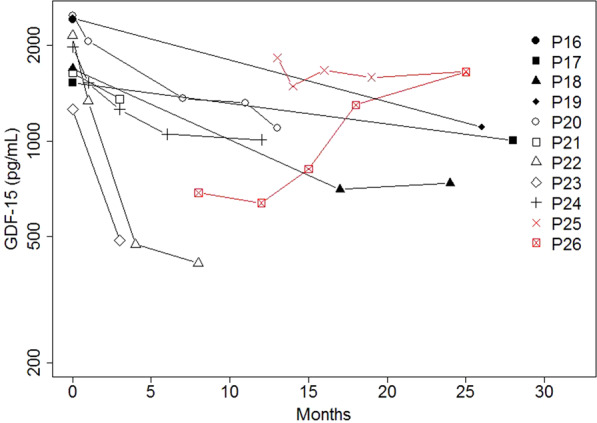
GDF-15 levels do not decrease over time in the absence of treatment. Development of serum GDF-15 over 28 months in treated patients (black lines) from group 2 versus untreated patients (P25 and P26, red lines).

The comparison between GDF-15 concentrations in treated adult patients and these two patients that stopped over a similar time frame (0 to 28 months) is shown in Fig. 3. While in the treated patients GDF-15 levels declined, in the two patients that stopped they went up or remained stable but did not lower in either case.

### Clinical outcome measures and correlation analysis

**Figure 4:**
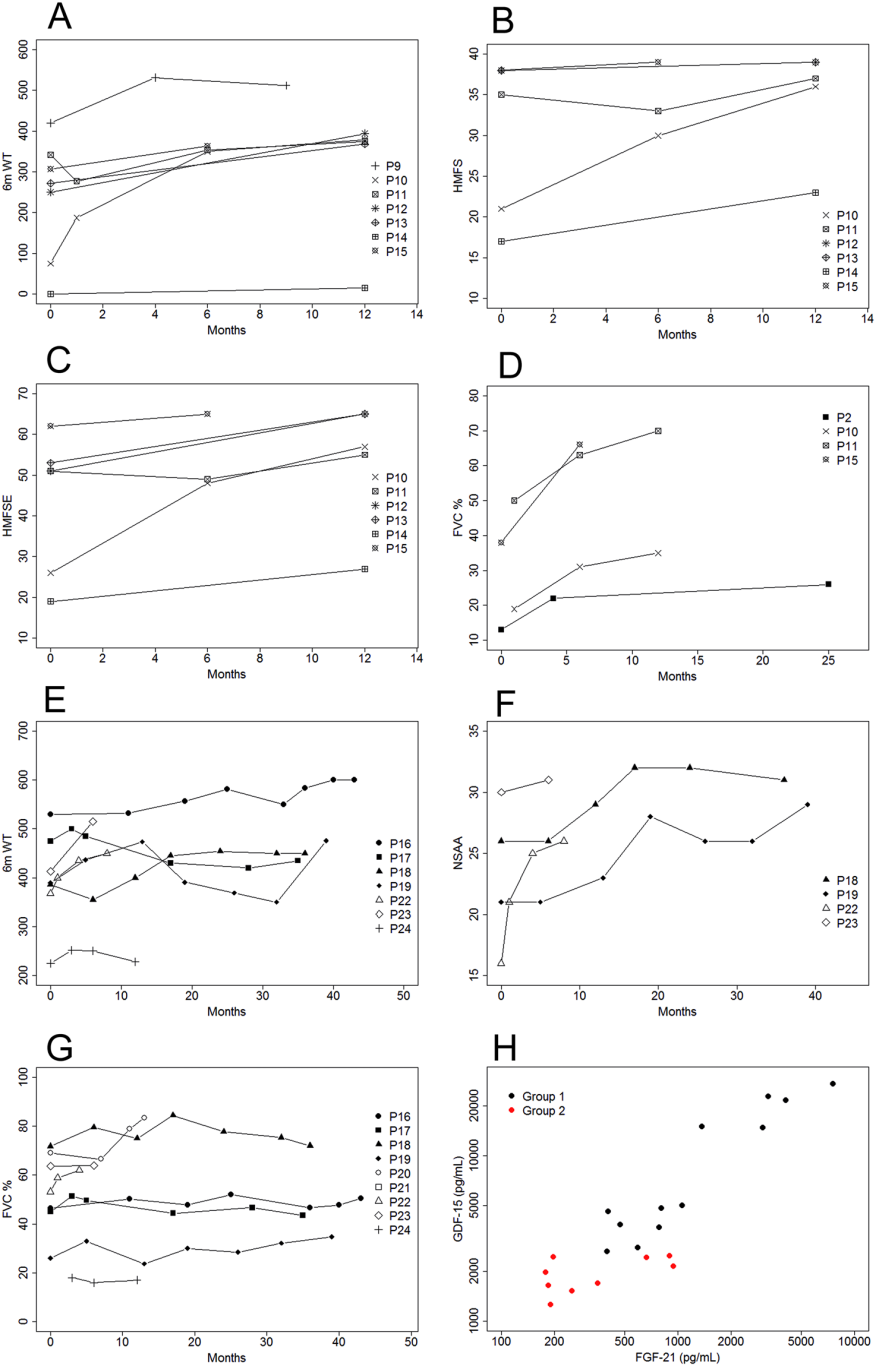
GDF-15 decrease was accompanied by improvement of several clinical outcome measures in treated patients. Development of selected functional tests for patients in group 1: 6MWT (**A**), HMFS (**B**), HMFSE (**C**), and % FVC (**D**) and for patients in group 2: 6MWT (**E**), NSAA (**F**), and % FVC (**G**) during treatment with deoxynucleosides. Correlation between basal levels of GDF-15 and FGF-21 in serum for group 1 (black) and group 2 (red) patients together (**H**).

We analyzed several clinical outcomes in both patients groups to determine whether the decline in GDF-15 was associated with clinical improvement following treatment. Tables 2 and 3 and Fig. 4 summarise this information.

Patients P1, P3, and P4 are the patients P5, P2, and P1 in our previous report^9^. They are in the early-onset and severe myopathy group in which the response to treatment was striking. This group of patients was defined by (1) onset before 24 months and (2) inability to walk, use of mechanical ventilation, or both within one year of onset. Without treatment, only 27.3% of patients described in the literature with such characteristics survived at least two years after onset (95% CI = 0.17–0.45)^9^. In contrast, with treatment, P1, P3, and P4 are still alive, four, six, and six years after disease onset respectively. One of these patients was weaned of ventilatory support and the two others regained the ability to walk without assistance. Patients P5, P6, and P7, not reported previously, also represent the most severe phenotype with early-onset and very rapid progression course. After treatment, they were evaluated with the Children’s Hospital of Philadelphia Infant Test of Neuromuscular Disorders (CHOP INTEND) assessments, which showed a mean improvement of 13.7 points after only three months of follow-up, and one (P7) even reached the maximum score on the scale three months later, which indicates complete motor recovery after only six months of treatment.

In childhood-onset cases, when we compared the latest post-treatment value (around one year after the start of the treatment) to the pre-treatment value for the more frequent outcome measures used, we found a significant improvement in the distance walked on the 6-minutes walking test (6MWT), with a mean increase of 105.8 meters (p = 0.01) (Fig. 4A). We also found a significant improvement in the scores of the Hammersmith Motor Function Scale (HMFS) and the Hammersmith Motor Function Scale-Expanded HMFSE scales (Fig. 4B,C), with an average improvement of 4.3 points and 12 points respectively between the pre-treatment and last post-treatment determinations (p = 0.03 in both cases) (Fig. 4A). Other clinical outcome measures (Egen Klassifikation Scale 2, EK2; North Star Ambulatory Assessment, NSAA) also reflected improvements, although without reaching statistical significance, when pre-treatment and post-treatment scores were compared. We did not have enough basal data regarding respiratory function (Forced Vital Capacity, FVC) to perform specific analyses in this group of patients (Fig. 4D).

In the adult group, consisting mainly of patients with late-onset disease, the 6MWT and the FVC showed a significant improvement when we compared pre- and post-treatment values (p = 0.05 and p = 0.04 respectively). This difference became more striking when we considered the changes in FVC during the first 12 months of treatment (p = 0.02). In particular, the 6MWT showed an improvement of an average of 53.2 meters after treatment (Fig. 4E), while the NSAA increased a mean of four points (Fig. 4F) and, more importantly, the mean FVC after treatment was 5% greater (Fig. 4G).

We had previously shown that mRNA and protein levels of GDF-15 and FGF-21 correlate strongly and significantly in children with a range of mitochondrial diseases and in myogenic cell lines in which mitochondrial damage was induced experimentally^12^. In keeping with this previous result, baseline and longitudinal concentrations of both factors also correlated with each other significantly in treated patients (p < 0.001; Spearman r = 0.843), (Fig. 4H).

When analysing group 1 and group 2 together, basal circulating GDF-15 and FGF-21 concentrations correlated significantly and negatively with age-at-treatment initiation (p = 0.006, r = −0.7 and p = 0.004, r = −0.7 respectively) and with weight expressed as BMI (p < 0.001, r = −0.8 and p = 0.024 and r = −0.5 respectively). In addition, basal GDF-15 concentrations correlated with the meters walked in the 6MWT (p = 0.003, r = −0.75).

### Inflammatory cytokines profile in patients with TK2 deficiency

Mitochondrial diseases and, in particular, TK2 deficiency are often associated with local (skeletal muscle) inflammatory responses which may be related to muscle cell damage or necrosis^12^. Given that GDF-15 can be induced by various stimuli related to inflammation, we assessed the circulating levels of the inflammatory cytokines IL-1, IL-6, IL-8, IL-15 and TNF (before and after treatment) in the eight patients from group 2 with late-onset phenotype that were followed in hospitals in Spain. We did not observe a significant increase in the average basal levels of any of the cytokines in either group of patients relative to the levels in age-matched controls nor a consistent pattern of change with time in treated patients (Supp. Fig. 2).

## Discussion

In previous work, our group described the global gene expression profile of skeletal muscle from patients with TK2 deficiency and identified the main molecular pathways implicated in the disease including the induction of GDF-15 expression partly under the control of p53. In addition, we demonstrated that GDF-15 outperformed other diagnostic biomarkers for mitochondrial diseases and postulated that it may also serve to monitor response to treatment based on preliminary data of one patient with TK2 deficiency that was treated with deoxynucleotides replacement therapy^14,15^. Since then, an expanded access program has shown a favorable side-effect profile and clinical efficacy of dNMP and deoxynucleoside therapies in patients with TK2 deficiency, some of them included in this study. Without any major side effects, the therapy had striking effects on early-onset severe myopathy patients, considerable functional improvements in childhood-onset cases and at least stabilization in late-onset ones^4^.

In the present work, we have assessed GDF-15 and FGF-21 in 24 patients with TK2 deficiency that started deoxynucleoside therapy either in their earlier years or as adults. The objective of this study was to ascertain the value of GDF-15 as a prognostic and treatment response biomarker for TK2 deficiency and compare it to FGF-21.

Together, the two groups of patients we analyzed represent the majority of patients with TK2 deficiency around the world that are treated. In addition, they encompass the known spectrum of the disease, from patients with early-onset and very rapid progression that is fatal in a few years without treatment, to childhood or late-onset cases with variable rates of disease progression, but also with bad prognosis because of respiratory involvement.

We confirm our previous findings that GDF-15 is markedly elevated in children and adults with TK2 deficiency^14,15^. GDF-15 levels before the start of treatment were elevated above the normal threshold value in all patients and on average six times higher in the paediatric patients compared to the adult patients, even more so in the children with early-onset and the most severe form of the disease. Similar results were obtained for FGF-21. Thus, both GDF-15 and FGF-21 are useful indicators of disease severity.

This was reflected in a significant and negative correlation between the basal concentration of both factors and the age of onset and/or treatment initiation so that the younger the children the higher the elevation of GDF-15 and FGF-21. Furthermore, pre-treatment GDF-15 levels were negatively associated with the meters walked on the 6MWT, one of the main indicators of motor function and also with body weight, expressed as the BMI. Thus, the higher the levels of GDF-15 (and FGF-21) the lower the BMI. Considering the evidence of the role of GDF-15 in the regulation of body weight and appetite^21, ^ one may speculate that the dramatically increased levels of circulating GDF-15 observed in these patients may be directly contributing to the rapid loss of body weight which is an important component of the disease but this requires additional studies.

Our results demonstrate that in patients treated with deoxynucleosides, GDF-15 concentrations decrease following a significant dependence over time and therefore GDF-15 fulfills the criteria for a treatment response biomarker. Although a similar trend was obtained for FGF-21 the decrease was not as consistent or as marked, particularly for patients in group 2. In contrast to treated patients, in TK2 deficient patients without treatment levels of GDF-15 tended to increase over short periods of time although more natural history data on untreated patients are necessary. All the patients under treatment showed important beneficial effects that are maintained over time or, at least, the stabilization of the disease. The response to treatment was greatest in group 1 (the paediatric group), but the mild motor improvements and respiratory stability shown in group 2 (the adult group) are a relevant clinical result as well since they show that treatment can reduce morbidity and mortality also in patients who started treatment in adulthood. In keeping with the extent of the clinical benefit, the rate of GDF-15 decrease was faster in children than in adults, in particular, in the most severe patients who are the ones that respond more markedly to the treatment in the form of extended survival, recovery from ventilatory support dependency, and regaining motor abilities. It has been recently reported that age-related metabolic changes in mice (namely increased deoxynucleoside catabolism and decreased anabolism with age) account, at least in part, for the limited efficacy of the treatment in this model^8^.

Both GDF-15 and FGF-21 are broad action cytokine/hormone that are altered at the same time in many disease states where they are routinely employed as diagnostic and prognostic biomarkers^12,20–25^. In particular, they are increasingly used individually or in combination as diagnostic biomarkers of mitochondrial diseases^9,16,18^.

In this study, we found that both GDF-15 and FGF-21 were elevated in all patients before deoxynucleoside treatment but that GDF-15 was more robust than FGF-21 to monitor changes over time in treated patients. Furthermore, GDF-15 correlated with motor function (6MWT) although this needs to be confirmed in a larger group of treated patients.

Our previous transcriptomic data of skeletal muscle indicates that p53 is the key regulator in a network of genes which are coordinately activated in response to TK2 deficiency leading to inflammation, activation of muscle cell death by apoptosis and induction of GDF-15 in muscle and serum. p53 plays a fundamental role in the control of the cell cycle, DNA repair and apoptosis in the nucleus and in mitochondria. Under certain conditions such as increased oxidative stress, p53 translocates to mitochondria to repair and/or promote replication of mtDNA^14^. Thus, our hypothesis is that mitochondrial DNA depletion due to TK2 mutations lead to activation of p53 which binds to the promoter region of GDF-15 inducing its expression and release by affected tissues.

Both GDF-15 and FGF-21 have a role in inflammation, for example, in pancreas, heart and adipose tissue^13,21–23^. Our data show that the profile of inflammatory cytokines was not consistently altered at baseline (not even in the children with the most severe early-onset phenotype) indicating that the elevated levels of GDF-15 and FGF-21 are not secondary to systemic inflammation. Similarly, treatment with deoxynucleosides did not result in a consistent pattern of change on the baseline levels of the inflammatory cytokines. However, we cannot exclude a contribution from local, tissue-specific, inflammation since we have previously observed over-expression of inflammatory markers in muscle biopsies from patients with TK2 deficiency and other mitochondrial DNA depletion syndromes^14^.

Limitations of the current study were that biochemical and clinical evaluations were performed at different time intervals depending on where the patients were being followed and that functional scales were not uniform across the cohort. This was difficult to overcome since in some cases samples had been collected retrospectively and not as part of a controlled study and some patients started treatment several years ago. Another important limitation is that we did not have the possibility to follow the levels of GDF-15 and FGF-21 in a group of untreated patients with TK2 deficiency in order to find out how they would change over time. TK2 deficiency is a very rare disease and given the severity of the phenotype and the effectiveness of deoxynucleoside treatment the vast majority of patients worldwide are being treated.

In conclusion, we propose that GDF-15 may be a potential biomarker of disease severity and response to treatment with deoxynucleosides in patients with TK2 deficiency and potentially also in other forms of mitochondrial DNA depletion and deletion syndromes where this treatment may also be beneficial.

## Materials and Methods

### Study design

We determined circulating GDF-15 and FGF-21 at baseline and at various time points in a group of 26 patients with mutations in TK2 treated with deoxynucleosides. We examined the rate of change over time, and correlated its basal and follow-up levels with various clinical parameters related to disease severity and response to treatment.

### Standard protocol approvals, registrations, and patient consents

This study was conducted in accordance with the Declaration of Helsinki and legal regulations and was approved by the Ethics and Research Committee of the Fundación Sant Joan de Déu. Written informed consent was obtained from all patients or their parents/guardians prior to enrolment.

### Study population and setting

Patients were included if they had two mutations in the TK2 gene and were receiving treatment with deoxynucleosides. Several of these patients have been described elsewhere^9^ and their basal characteristics are detailed in Table 1 and Supplementary Table S1. Patients were followed in five Hospitals in Spain (Hospital 12 de Octubre, Madrid; Hospital Sant Joan de Déu, Barcelona; Hospital de la Santa Creu I Sant Pau, Barcelona; Hospital Virgen del Rocio, Seville and Hospital Universitario Donostia, San Sebastian) and in the Columbia University Medical Center (New York, USA).

### Oral administration of deoxynucleosides

All patients were treated with oral doses between 300–400 mg/Kg/day of each nucleoside (thymidine, dThd, and deoxycytidine, dCtd). Two patients (P3 and P4) started with nucleotides (dTMP and dCMP) and later switched to nucleosides (in a one-to-one ratio of height:weight), once it became clear that these are the active agents^5,6^. The majority of patients (n = 24) are currently being treated with dThd and dCtd. Two adult-onset individuals (P25 and P26) had to stop treatment because of liver enzymes elevation and were excluded from the main analyses.

### Analytical methods

Serum samples were collected, allowed to clot, centrifuged at 1300 g for 10 minutes, aliquoted, and stored at −80 °C until the moment of the analysis. Long-term storage of samples at −80 °C does not affect GDF-15 or FGF-21 stability according to our experience and published data^20^.

Previously, we had compared serum (collected in serum separator tubes) and plasma samples (collected in EDTA, heparin lithium or heparin sodium tubes) from the same individuals and found comparable GDF-15 and FGF-21 levels regardless of the type of sample and collection method. We also compared different times of sample collection (fasting versus postprandial) and found no significant differences for either GDF-15 or FGF-21 (data not shown).

Quantitative sandwich ELISAs were performed using the Quantikine Human GDF-15 Immunoassay kit (R&D Systems, Minneapolis, USA) and the FGF-21 ELISA KIT (R&D Systems, Minneapolis, USA or Millipore, Massachusetts, USA) as previously described^9,15^. Sensitivity, intra-assay and inter-assay variances (expressed as CV%) are 4.39 pg/mL and 8.69 pg/mL, 2,26% and 3,43% and 5,43 and 7,5% for GDF-15 and FGF-21 respectively.

Briefly, serum samples were either diluted ¼ in the sample diluent buffer provided with the kit as recommended by the manufacturer (GDF-15) or used undiluted (FGF-21). A volume of 50ul of diluted serum sample was added to each well and assays were performed in duplicate. A standard curve was prepared using a dilution series (in pg/mL) of a recombinant human GDF-15 or FGF-21 standard diluted in the sample diluent buffer provided with the kit. To determine the optical density of the preparations we used a microplate reader (Molecular Probes) set to 450 nm subtracting readings at 540 nm for wavelength correction. The values from each sample were extrapolated from a standard four-parameter logistic curve using the SoftMax software of the microplate reader. Final concentrations were corrected by the dilution factor.

Interleukin (IL)-1B, IL-6, IL-8, IL-15, tumor necrosis factor (TNF) and monocyte chemoattractant protein-1 (MCP1) were quantified using a multiplex analysis system based on fluorescently labeled microspheres linked to specific antibodies (HCYTOMAG-60K-06, Millipore Linco Research/Millipore, Massachusetts, USA) using a Luminex100ISv2 instrument.

### Outcome measures

Motor assessment: Patients underwent periodic motor assessments with at least one of the following: 6-minute walk test (6MWT); North Star Ambulatory Assessment (NSAA), which evaluates motor goals with a score range of 0–34 as values of minimum and maximum motor skills, respectively; Egen Klassifikation (EK2), which evaluates functional capacity in nonambulatory patients with a score range of 30–0 as values of minimum and maximum functional capacity, respectively; the Children’s Hospital of Philadelphia Infant Test of Neuromuscular Disorders (CHOP INTEND) used to evaluate the motor skills of infants with SMA with a score range from 0 to 64, with higher scores indicating better motor function; Hammersmith Motor Functional Scale (HFMS), which assesses the physical abilities of children with non-ambulant Spinal Muscular Atrophy, with a total score achievable of 40, with higher scores reflecting better physical abilities; or the Hammersmith Motor Functional Scale Expanded (HFMSE), which contains additional items for the ambulant population, with a total score achievable of 66.

Respiratory evaluation: We measured forced vital capacity (FVC) and maximal inspiratory pressure (MIP) in an upright position in compliant patients.

### Statistical analysis

Descriptive statistics and correlation analyses were performed using R version 3.2 and GraphPad PRISM version 8.1.2 A. A p-value of < 0.05 was considered statistically significant. Spearman’s correlation was used to determine the relationship between numerical variables, and a paired non-parametric test was used to compare variables at two times (pre- versus post-treatment) for the same patient. Repeated measures mixed linear models were used to determine whether numerical variables changed over time if data was available at multiple time points.

## Supplementary information

Supplementary information.

## Data Availability

The raw data and protocols used for this study are available from the corresponding author.
