# Supplementary material for: Growth Differentiation Factor 15 is a potential biomarker of therapeutic response for TK2 deficient myopathy
Source: Sci Rep. 2020 Jun 22;10:10111. doi: 10.1038/s41598-020-66940-8 (PMC7308386; doi:10.1038/s41598-020-66940-8)
Supplement: Supplementary file 1 — Supplementary information. [file 41598_2020_66940_MOESM1_ESM.docx]

**Growth Differentiation Factor 15 is a potential biomarker of therapeutic response for TK2 deficient myopathy.**

Cristina Dominguez-Gonzalez, MD^1,2,3^, Carmen Badosa^4^, Marcos Madruga-Garrido^5^, MD, Itxaso Martí^6^, MD, Carmen Paradas^7,8^, PhD, Carlos Ortez^4^, MD, Jordi Diaz-Manera^3,9^, PhD, Andres Berardo^10^, PhD, Jorge Alonso-Pérez^9^, MD, Selena Trifunov^4^, PhD, Daniel Cuadras^11^, PhD, Susana G. Kalko, PhD^12^, Cora Blázquez-Bermejo^3,13^, Yolanda Cámara^3,13^, PhD, Ramon Martí^3,13^, PhD, Fabiola Mavillard-Saborido^7,8^, MD, Miguel A. Martin^2,3^, PhD, Julio Montoya^3,14^, PhD, Eduardo Ruiz-Pesini^3,14^, PhD, Joan Villarroya^15,16^, PhD, Raquel Montero^3,17^, PhD, Francesc Villarroya^15,16^, PhD, Rafael Artuch^3,17^, PhD, Michio Hirano^10^, PhD, Andrés Nascimento^3,4,^, MD Cecilia Jimenez-Mallebrera*^3,4,18^, PhD.

^1^Neurology Department, Neuromuscular Disorders Unit, 12 de Octubre Hospital, Madrid, Spain.

^2^Research Institute i+12, 12 de Octubre Hospital, Madrid, Spain.

^3^Biomedical Network Research Centre on Rare Diseases (CIBERER), Instituto de Salud Carlos III, Madrid, Spain.

^4^Neuromuscular Unit, Neuropediatrics Department, Institut de Recerca Sant Joan de Déu, Hospital Sant Joan de Déu, Barcelona, Spain.

^5^  Neuromuscular Disorders Unit, Neuropediatrics Department, Instituto de Biomedicina de Sevilla, Hospital Universitario Virgen del Rocío, Consejo Superior de Investigaciones Científicas, University of Seville, Seville, Spain.

^6^ Neuropediatrics Department, Hospital Universitario Donostia, San Sebastian, Spain.

^7^Neuromuscular Disorders Unit, Neurology Department, Instituto de Biomedicina de Sevilla, Hospital Universitario Virgen del Rocío, Consejo Superior de Investigaciones Científicas, University of Seville, Seville, Spain.

^8^ Biomedical Network Research Centre on Neurodegenerative Diseases (CIBERNED), Instituto de Salud Carlos III, Madrid, Spain.

^9^Neuromuscular Diseases Unit, Hospital de la Santa Creu i Sant Pau, Universitat Autònoma de Barcelona, Barcelona, Spain.

^10^Department of Neurology, Columbia University Medical Center, New York, USA.

^11^ Statistics Unit, Fundación Sant Joan de Déu, Barcelona, Spain.

^12^Moebius Research Ltd, Systems Biomedicine, London

^13^Research group on Neuromuscular and Mitochondrial Diseases, Vall d’Hebron Research Institute, Universitat Autònoma de Barcelona, Barcelona, Spain.

^14^Departmento de Bioquímica y Biología Molecular,  Universidad de Zaragoza, Instituto de Investigación Sanitaria de Aragón (IIS-Aragón), Zaragoza, Spain.

^15^ Biochemistry and Molecular Biology Department^,^ Institute of Biomedicine (IBUB), Institut de Recerca Sant Joan de Déu, University of Barcelona, Spain.

^16^Biomedical Network Research Centre on Obesity and Nutrition (CIBEROBN), Instituto de Salud Carlos III, Madrid, Spain.

^17^Clinical Biochemistry Department, Institut de Recerca Sant Joan de Déu, Hospital Sant Joan de Déu, Barcelona, Spain.

^18^ Genetics Department, Faculty of Biology, University of Barcelona, Barcelona, Spain.

*corresponding author

Cecilia Jiménez-Mallebrera, PhD. Neuromuscular Unit, Institut de Recerca Sant Joan de Déu, Hospital Sant Joan de Déu, Calle Santa Rosa 39-57, Esplugues de Llobregat, Barcelona 08950, Spain. Tel: + 34 936009751; Fax: + 34 936009771. cjimenezm@fsjd.org.

Table S1: List of the relationship between the patients in the current study and in previous publications

| **ID** | **PREVIOUSLY REPORTED** |
| --- | --- |
| 1 | P5 ^9^ |
| 2 | P9 ^9^ |
| 3 | P2 ^9^ |
| 4 | P1 ^9^ |
| 5 | Not reported |
| 6 | Not reported |
| 7 | Not reported |
| 8 | Not reported |
| 9 | Not reported |
| 10 | Not reported |
| 11 | Not reported |
| 12 | Not reported |
| 13 | Not reported |
| 14 | Not reported |
| 15 | Not reported |
| 16 | P1 ^4^; P13 ^9^ |
| 17 | P5 ^4^; P15 ^9^ |
| 18 | P3 ^4^; P16 ^9^ |
| 19 | P2 ^4^; P14 ^9^ |
| 20 | P4 ^4^ |
| 21 | P12 ^4^ |
| 22 | Not reported |
| 23 | P16 ^4^ |
| 24 | Not reported |
| 25 | P8 ^4^ |
| 26 | P6 ^4^ |

Figure S1: Changes in GDF-15 and FGF-21 grouped by fixed intervals before and after treatment initiation.


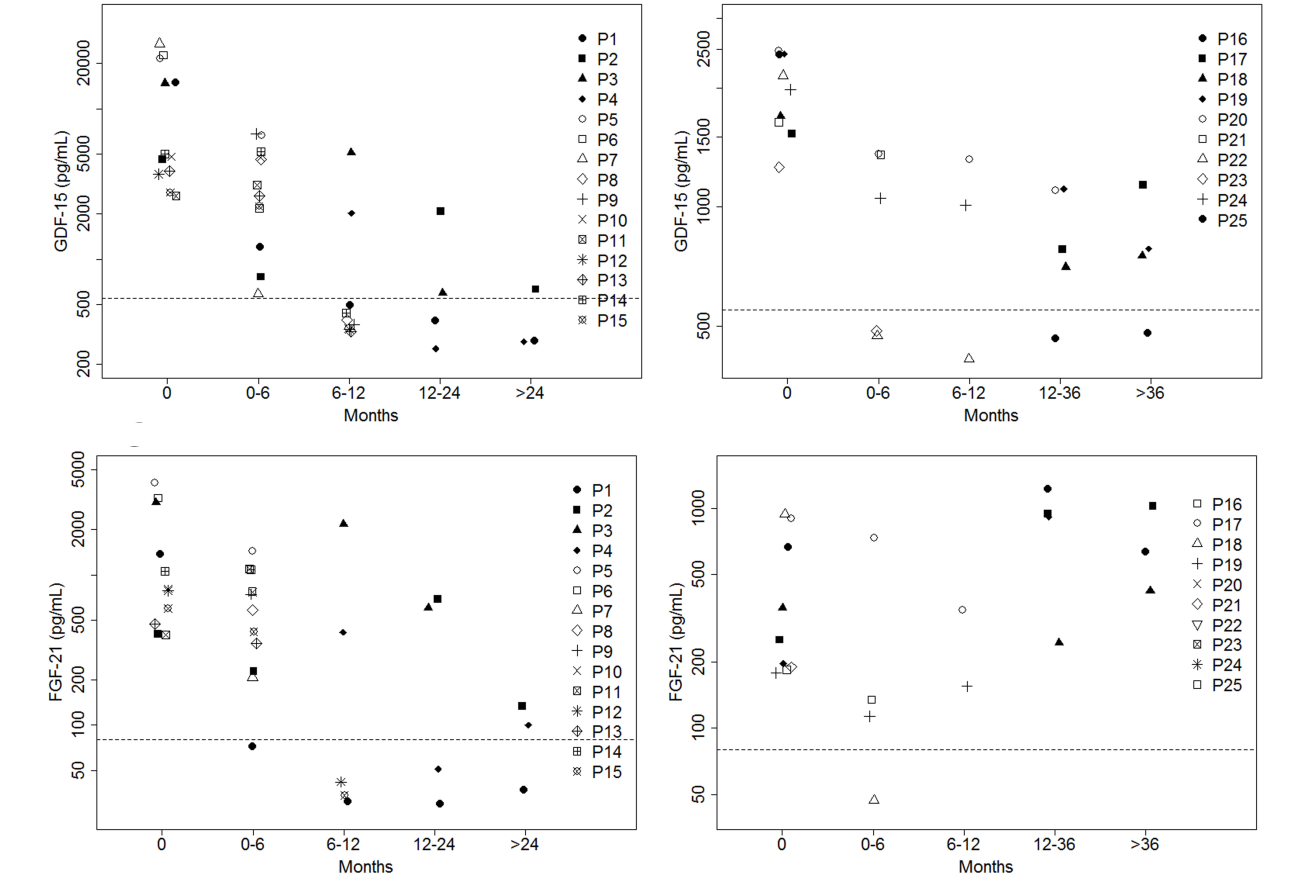


**Legend**

Figure S1: Changes in GDF-15 (Top) and FGF-21 (Bottom) grouped by fixed intervals before and after treatment initiation in Group 1 (Left) and Group 2 (Right) patients.

Figure S2: Development of inflammatory cytokines in treated adult patients


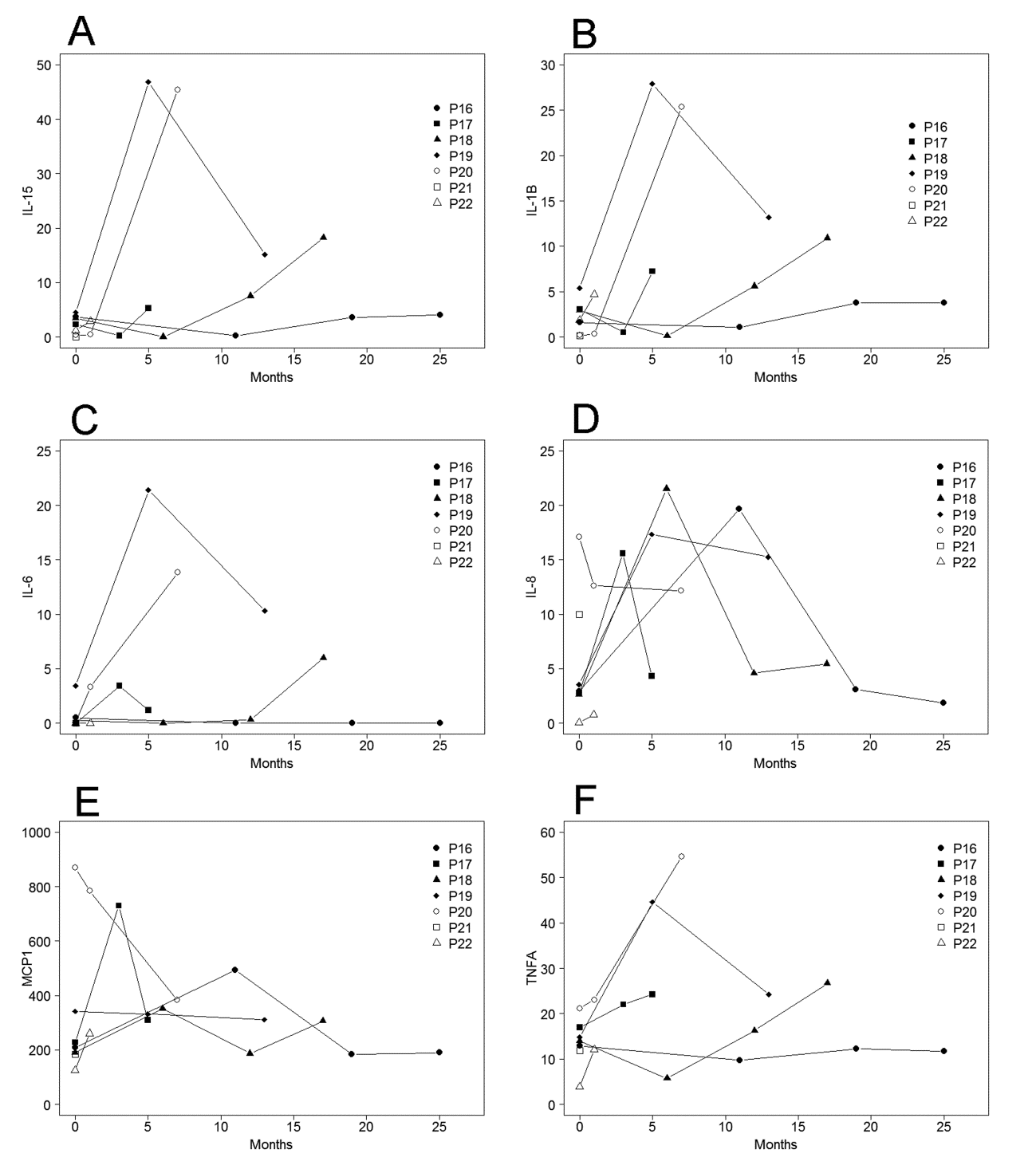


**Legend**

Figure S2: Analysis of inflammatory cytokines in treated patients from group 2.: IL-15 (A); IL1-B (B); IL-6 (C); IL-8 (D); MCP1 (E) and TNF-α (F).
